# Small intestine transplant immunologic risk assessment: More data is needed

**DOI:** 10.1016/j.intf.2025.100061

**Published:** 2025-05-27

**Authors:** J.M. Ladowski, Mariya L. Samoylova, Jeffrey Ord, Annette M. Jackson, Debra L. Sudan

**Affiliations:** Division of Division of Abdominal Transplant Surgery, Department of Surgery, Duke University, Durham, NC, United States

**Keywords:** Intestinal transplant, Intestinal transplant outcomes, International intestinal transplant registry, Eplets, HLA Matchmaker

## Abstract

**Background:**

Intestinal transplantation (IT) remains the only treatment modality for patients with irreversible intestinal failure who cannot be maintained on chronic parenteral nutrition. Multiple reports have demonstrated an association between donor-specific antibodies (DSA) in IT graft rejection, however these studies have largely been single-center analyses. The goal of this study was to examine the available immunological data from the International Intestinal Transplant Registry (IITR) and evaluate the association with rejection.

**Materials and methods:**

Demographic, outcomes, and serologic HLA data from donor/recipient pairs was obtained from the IITR. Outcomes including rejection, graft survival, and patient survival were analyzed based on age, DSA reporting, and molecular HLA mismatch. Eplet mismatch analysis was performed using HLAMatchmaker.

**Results:**

Our analysis revealed significant limitations in the granularity of data contained in the IITR.

**Conclusion:**

The IITR database is a promising option to perform a multicenter analysis of IT outcomes, but there are limitations in the data to allow a thorough immunologic examination.

## Introduction

Intestinal transplant (IT) is infrequently performed in the United States, with only 96 transplants in 2021, but remains the primary treatment option for patients with irreversible intestinal failure who have failed parenteral nutrition (PN) [1]. Since inception, substantial progress has been made in graft survival following IT with 5-year survival of 46 % and 58 %, respectively for intestine vs. liver-intestine recipients [2]. Much of this progress is owed to optimization of immunosuppressive regimens, yet the percentage of recipients experiencing an episode of graft rejection remains high and is one of the most significant barriers to long-term graft and patient survival [3], [4], [5]. Further investigation is needed therefore into the mechanism of rejection in intestinal transplantation, especially in terms of the impact of HLA matching and development of donor specific antibody considering recent findings in other solid organ transplants.

The International Intestinal Transplant Registry (IITR) has collected data on the outcomes of ITs since 1985 [6]. The data, maintained by The Transplantation Society, is typically published biannually. Prior reports have demonstrated improving patient and graft survival in intestinal transplant recipients [6], [7]. Post-transplant DSA data was added to the data forms starting in 2010, and theoretically provides an opportunity for a multi-institutional analysis of this important immunological factor.

The human leukocyte antigen (HLA) protein is the primary donor alloantigen recognized by the recipient immune system in ABO-compatible transplants. The replacement of serological HLA typing methods with DNA based methods in the 1990’s significantly advanced our understanding of HLA polymorphism and aided in our assessments of HLA histocompatibility [8], [9]. Broader implementation of HLA sequencing to provide allele level or high-resolution HLA typing is permitting more sophisticated HLA mismatch assessments between recipient and donor pairs. Multiple bioinformatic tools have been developed for assessing HLA molecular mismatch; some count amino acid differences between recipient/donor alleles while others examine the physiochemical quality of the amino acid mismatch (size and charge) [10]. HLA Matchmaker, one of the first tools available, distinguishes short polymorphic sequences (eplets) that correlate with HLA antibody patterns [11].

Studies in renal transplantation demonstrate a nearly linear correlative relationship between increasing HLA mismatch score and poor graft outcomes. A lower molecular mismatch score, particularly with regards to class II HLA, is associated with decreased incidence of T cell mediated rejection and *de novo* donor-specific antibody (dnDSA) development [12], [13], [14]. Studies in lung, heart, pancreas, and liver transplant have also provided some evidence for this relationship in other solid-organ transplants [15], [16], [17]. Recent small single center studies in intestine transplantation also suggest an association between dnDSA development and increased rates of acute and chronic rejection, and graft failure [5], [18], [19], [20], [21].

The significance of dnDSA development and the association of DSA with rejection in intestinal transplant has not been clearly established. The diagnostic criteria of antibody-mediated rejection in IT is not universally accepted and where described, relies on a combination of serologic, clinical, and histologic data [4], [22]. Several prior single center studies have inconsistent findings and are limited by small sample size and inclusion of both isolated intestine and liver containing multi-visceral allografts, which may confound analyses due to a potential immune-protective effect of a liver component.

The goal of this study was to examine the immunologic risk associated with DSA development and HLA molecular mismatch in intestine transplant recipients, from an international multi-institutional dataset. Our hypothesis was that traditional solid organ immunological risk factors (occurrence of rejection, dnDSA and HLA eplet mismatch scores) would correlate with worse IT outcomes.

## Materials and methods

### Patient population

This retrospective study was determined to be exempt by the Institutional Review Board (Protocol #00102947). The IITR was queried for all intestinal transplants performed between 2011 and 2019 for entries with inclusion of data regarding DSA. Donor and recipient characteristics, including induction and maintenance immunosuppression agents were recorded.

### Graft definitions

Graft types as defined by Intestinal Rehabilitation and Transplantation Association (IRTA) included: 1) small bowel alone 2) small bowel and liver 3) multi-visceral transplant (MVT) 4) modified multi-visceral transplants (mMVT).

### Outcome definitions

The primary endpoint of this study was to determine the impact of DSA on the occurrence of rejection, graft survival and patient survival after IT. An important secondary focus was to determine the impact of EpMM on the development of DSA after intestine transplantation. Due to the potential for multiple factors that could confound our analysis, we first examined the impact of graft type, recipient age, occurrence of rejection and development of DSA independently for their potential impact on graft and patient survival.

Graft failure was defined as a return to at least supplemental total parenteral nutrition (TPN) as reported in the database. Development of dnDSA was defined as DSA reported in the IITR database after IT in recipients that did not have identified pre-transplant DSA. Rejection was reported in the IITR database as either occurring during the hospitalization or after hospitalization, when available the date of the rejection episode was used. Biopsies were performed either for cause or per hospital protocol, it is unknown if the clinician knew the status of DSA at the time of biopsy.

### HLA typing

Low-resolution and, when available, high-resolution, molecular typing of the donor and recipient HLA were obtained from the IITR. This data routinely included typing for HLA-A, -B, -C, -DRB1, and –DQB1 antigens. High resolution HLA alleles were imputed using the National Bone Marrow Donor Program’s Haplostats software (www.haplostats.org) and HLA linkage disequilibrium analysis (DRB3/B4/B5); exclusions were made if insufficient typing made accurate predictions impossible. High-resolution HLA alleles, either predicted from Haplostats or reported in the database, were entered into the HLA-Matchmaker software (HLA-Matchmaker-A, B, C and -DR, DQ, DP matching v3.0, www.epitopes.net), and eplet-mismatches (EpMM) between donor-recipient HLA antigen pairs were calculated. EpMM are characterized as either all EpMM, antibody-verified EpMM, or non-antibody verified EpMM. Given the potential increased immunogenicity of antibody-verified eplets compared to non-antibody-verified, antibody-verified EpMM were chosen for outcome-related analysis [20].

### Statistics

All statistical and univariate analysis was performed in GraphPad Prism 9 (GraphPad Software, La Jolla, CA). Non-normally distributed variables were analyzed with an unpaired Mann-Whitney-Wilcoxon non-parametric, two-sided test with significance set at p ≤ 0.05. Kaplan-Meier survival analysis based on freedom from all-cause and all-time rejection, graft survival, and patient survival were created and analyzed using the Gehan-Breslow-Wilcoxon test for significance. Patients lost to follow-up without evidence of rejection, graft failure, or death respectively were censored at the time of last known follow-up. Graphs of these Kaplan-Meier survival curves were made in GraphPad Prism 9.

To evaluate the impact of EpMM scores on post-transplant DSA development, incidence of rejection, graft failure, and patient survival, deciles were calculated for each of the examined HLA groups (antibody-verified HLA class I (A, B, C), class II (-DRB1/3/4/5 and DQA1/DQB1), HLA-DRB1/3/4/5, and HLA–DQA1/DQB1). The highest and lowest 20th percentile were chosen for analysis to approximate a standard deviation outside the mean.

## Results

### Principal findings

Our hypothesis was that a significant HLA eplet mismatch between donor and recipient would be associated with worse graft outcomes in IT. We sought to use the IITR, an international data registry, to analyze the results in an international cohort. Our principal finding (detailed below) is that there are deficiencies in the IITR, both in data granularity and completeness, that limit the ability to complete analyses regarding the immunologic potential of rejection.

### Limitations in reporting

The IITR database was queried for recorded transplants with information on DSA performed between 2011 – 2019, providing 228 records in total. This represents 21 % of the reported 1065 ITs performed during the same time period in the United States alone [1]. Of the 228 IT records, 121 patients (53 %) were excluded from analysis due to lack of donor or recipient HLA typing (n = 31, 14 %), lack of granular DSA information (n = 62, 27 %), preformed DSA (n = 9, 4 %), death within 24 h post-operatively (n = 13, 6 %), and insufficient HLA-DQ typing such that accurate imputation of HLA class II alleles could not be performed (n = 6, 3 %). The analysis cohort therefore consisted of 107 patients. Only 55 of the original 107 recipient/donor pairs (51 %) reported high-resolution typing for either the donor or recipient.

### Available patient demographics

**Fig. 1:**
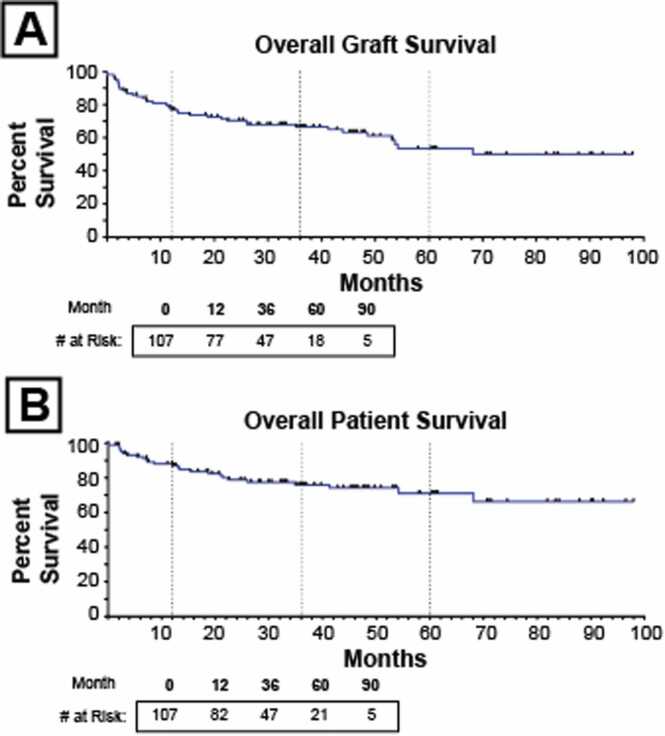
Kaplan-Meier survival curves depicting A) graft survival and B) patient survival for the studied population of 107 patients. Vertical dotted lines represent the 12-month, 36-month, and 60-month time points.

The characteristics of the available donor and recipient age, gender, ABO compatibility, country of transplant, indication for transplant and immunosuppressive medications are summarized in Supplementary Table 1. The median follow-up time was 33 months (mean 35 months). The majority of transplants were performed in the United States (81 %) for short gut (65 %) (Supplementary Table 1). Forty patients (37.4 %) experienced graft failure at a median time of 10 months after IT (mean 17 months). The 1-, 3-, and 5-year graft survival was 77 %, 67 %, and 54 % respectively (Fig. 1A). Death occurred in 25 patients (23 %) at a median time of 12 months after IT (mean 17 months). The 1-year, 3-year, and 5-year patient survival for the cohort was 87 %, 76 %, and 71 % (Fig. 1B).

### Reported rejection, DSA, and HLA mismatch data

The IITR’s current method of data capture for rejection is to record the first episode, severity, treatment, and the corresponding date. Rejection was reported in 71 % of IT recipients (n = 76), with an average onset of ∼1 month post-transplant (n = 40, median 0.6 months, range 0.2 – 13 months). We detected no differences in graft or patient survival identified based on occurrence of rejection in this group of IT recipients (Supplementary Figure 1A, Kaplan-Meier graft survival, p = 0.11 and Supplementary Figure 1B, Kaplan-Meier patient survival p = 0.38)

During the study period, the incidence of dnDSA in IT recipients was 33 %, (n = 35). For those with reported time from transplant to DSA development, the median time to DSA development was 17 days (range 4 days to 1100 days, n = 28). In 33 patients with available specificity of reported DSA: 2 were directed against class I HLA alone, 21 were directed against class II alone, and 10 were directed against both class I and class II. In this study, there was no difference identified in incidence of any rejection occurrence in recipients with development of dnDSA (27/35; 77 %) compared to those without dnDSA (49/72; 68 %, p = 0.37) (Supplementary Table 2). This is further confirmed by the Kaplan-Meier graft and patient survival curves based on the presence or absence of dnDSA, shown in Supplementary Figure 2.

HLA Eplet mismatch (EpMM) scores were examined for the entire cohort of 107 IT recipients (Supplementary Table 3). The median antibody-verified EpMM score for HLA class I (A, B, C) was 10 (range 0 – 16, mean 10) and for composite class II (-DRB1/3/4/5 and DQA1/DQB1) the median EpMM score was 10 (range = 0 – 49, mean 12. Further delineation for antibody-verified HLA class II EpMM scores revealed a median score of 5 (range 0 – 41, mean 8) for HLA-DRB1/3/4/5 and 4 (range 0 – 9, mean 4) for HLA–DQA1/DQB1. Comparing the highest and lowest quintiles of antibody-verified EpMM scores, there was no significant difference in dnDSA development, occurrence of rejection, graft survival, or patient survival (Supplementary Table 4).

## Discussion

Our focus was to use to a multicenter registry to determine the impact of dnDSA and EpMM on the occurrence of IT rejection. The IITR was chosen due to prior reports of > 95 % capture rate of the world’s cases of ITs. Additionally, HLA typing and DSA development are not required to be reported by UNOS for intestine transplant candidates and recipients and the IITR would therefore be the only multi-institutional source of this information. We ultimately found that there were deficiencies in the reporting of outcomes to the IITR to dnDSA development, timing of development, rejection episodes, timing of rejection, and HLA typing.

There is a possibility that we did not detect a correlation between DSA/EpMM and rejection, graft survival, and patient survival because of a real difference in mechanism of rejection of intestine allografts. A potential explanation could be that the transplanted intestinal tissue, with its large immune cell burden, may be more immunogenic than other solid organs, and elicits a more robust and earlier immune response, although this is conjecture [23]. This may be somewhat supported by the markedly higher incidence of rejection episodes after IT (50–70 % in the first year), compared to other solid organ allografts (e.g. 6.8 % in kidney recipients and 11.5 % among adult liver recipients [24], [25].

It is also noteworthy that the median-time to detection of dnDSA in this IT population was 17 days, which is similar to other reports but starkly different than other solid-organs where the average time to dnDSA development is greater than 10 months and 52 days, respectively [3], [5], [19], [20], [21], [26]. The rapid time to reported *dn*DSA appears short in a highly immunosuppressed IT recipient, since an immunocompetent model requires approximately 14 days for a de novo antibody response to a new antigen stimulus and would be expected to be delayed in the profoundly immunosuppressed IT recipient [27]. The dnDSA reported here (and by others after IT) may be in fact a memory response due to unrecognized, undetected, or unreported pre-transplant HLA sensitization. Future studies that incorporate historic episodes for potential sensitization, HLA Ab testing, or serial post-operative HLA Ab testing would be useful to further assess this possibility.

A lack of correlation between DSA/EpMM and post-transplant outcomes would be surprising given the substantial evidence for EpMM score predicting dnDSA development and rejection in other solid organs and previous IT recipients [5], [18], [28], [29], [30]. We attribute our findings to limitations in the number of reported ITs containing DSA data - our query contains less than 23 % of ITs performed in the US alone. We also cannot exclude that potential reporting and survival biases might impact the data collection - 81 % of reported data are from the US. Although we found a similar incidence of rejection during initial hospitalization (∼39 %), overall rejection (70 %), and post-transplant dnDSA compared to previously published reports, we also found improved long-term 5-year graft survival (71 vs 49 %) and patient survival (77 vs 58 %) [2], [3], [5], [6], [28], [31]. While this could reflect an improvement in the management of ITs recipients (as outcomes related to IT are continuously improving), it warrants further query.

## Conclusion

The IITR represents the best opportunity to evaluate the role of DSA and EpMM on outcomes in a multi-national sample of IT recipients and since 2015, the IITR now collects information regarding HLA matching, DSA status, and immunosuppressive strategies. Inclusion of DSA status at the time of transplant would further clarify the immunologic risk the recipient faces post-transplant [32], [33]. Although data is absent prior to 2015, one solution could be that the IITR database be combined with others such as UNOS, where complete donor HLA typing for example would be anticipated to be present in any intestine donor that also donated a renal allograft. We hope future studies will be possible with a larger volume of IT recipients and more complete DSA monitoring and HLA typing to determine the true immunologic impact of EpMM on DSA and rejection after IT. It is the author’s hope that this report may serve as a call to centers to report ITs and to the IRTA scientific committee to identify mechanisms to link the data from multiple sources to eliminate the burden of repeated data entry and provide data in a timely fashion.

## CRediT authorship contribution statement

**Ladowski Joseph:** Writing – review & editing, Writing – original draft, Validation, Supervision, Software, Methodology, Investigation, Formal analysis, Conceptualization. **Samoylova Mariya:** Writing – review & editing, Formal analysis, Data curation, Conceptualization. **Jeffrey Ord:** Writing – review & editing, Writing – original draft, Formal analysis, Data curation, Conceptualization. **Jackson Annette:** Writing – review & editing, Writing – original draft, Validation, Supervision, Methodology, Formal analysis, Conceptualization. **Sudan Debra:** Writing – review & editing, Writing – original draft, Validation, Supervision, Methodology, Investigation, Formal analysis, Conceptualization.

## Ethical statement

This is a retrospective study and no HIPPA related information was utilized.

## **Patient consent**

This is a retrospective study and no patient consent was needed.

## Disclosures

A.M.J. has received research support from 10.13039/100016477CareDx, Inc. and has received consulting honoraria from Hansa Biopharma and Novartis. The remaining authors have no other disclosures.

## Funding

There are no pertinent funding sources to disclose.

## Declaration of Competing Interest

The authors declare no conflicts of interest to disclose.
