# Supplementary material for: Small intestine transplant immunologic risk assessment: More data is needed
Source: Intest Fail. 2025 May 27;5:100061. doi: 10.1016/j.intf.2025.100061 (PMC12851323; doi:10.1016/j.intf.2025.100061)

**Supplementary Figure 1:** Kaplan-Meier survival curves depicting graft survival and patient survival for the studied population of 107 patients based on rejection (Figures A, p = 0.11, and B, p = 0.38). Vertical dotted lines represent the 12-month, 36-month, and 60-month time points.

**
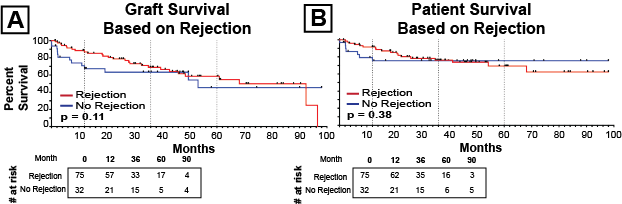
**

**Supplementary Figure 2:** Kaplan-Meier survival curves depicting graft survival and patient survival for the studied population of 107 patients based on DSA presence (Figures A, p = 0.56, and B, p = 0.53) Vertical dotted lines represent the 12-month, 36-month, and 60-month time points.

**
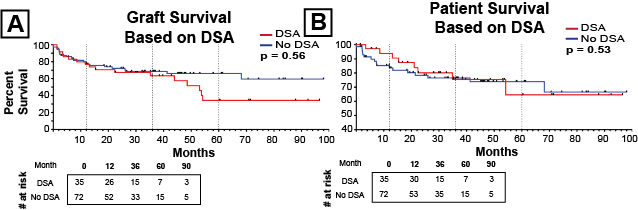
**

**Supplementary Table 1:** Summary of the available characteristics of the donor, transplant and recipient. Where indicated the results column demonstrate either the median with range of reported values or raw number of reported values. BMI is expressed as an average with the range in parentheses. Note some groupings may not equal study sample of 107 if data was not reported.

| **Characteristic** | **Overall (n = 107)** |
| --- | --- |
| **Recipient Age (years)** | 8.5 (0.04 - 57.0) |
| **Recipient Gender** |  |
| Male | 66 (62%) |
| Female | 41 (38%) |
| **Recipient BMI** | 18.92 (12.44 - 32.64) |
| **Re-transplant** |  |
| Yes | 11 (10%) |
| No | 96 (90%) |
| **Country of Transplant** |  |
| United States | 87 (81%) |
| Belgium | 8 (8%) |
| France | 7 (7%) |
| Czech Republic | 2 (2%) |
| Canada | 2 (2%) |
| Australia | 1 (1%) |
| **Donor Age (years)** | 7.0 (0.0 - 43.0) |
| **ABO compatibility** |  |
| Identical | 74 (69%) |
| Compatible | 21 (20%) |
| Not available | 12 (11%) |
| **Indication for Transplant** |  |
| Short gut | 70 (65%) |
| Mucosal Defect | 11 (10%) |
| Motility Disorder | 21 (20%) |
| Malignancy | 4 (4%) |
| **Induction Immunosuppression** |  |
| Alemtuzumab | 3 (3%) |
| Anti-thymocyte globulin (ATG) | 61 (57%) |
| Daclizumab or Basiliximab | 32 (30%) |
| Bortezomib | 1 (1%) |
| Other (tacrolimus, methylprednisone, donor-specific transfusion) | 11 (10%) |
| **Maintenance Immunosuppression** |  |
| Tacrolimus | 87 (81%) |
| *Mycophenolate mofetil (MMF) | 34 (32%) |
| Prednisone | 80 (75%) |
| Rapamycin | 3 (3%) |
| Azathioprine | 10 (9%) |
| *Tacrolimus + Prednisone | 78 (73%) |
| *Tacrolimus, Prednisone, + MMF | 29 (27%) |

**Supplementary Table 2:** Number of patients with and without new reported DSA experiencing rejection (n = 76), graft loss (n = 40), and mortality (n = 25). The p-values of a Mann-Whitney non-parametric t-test between the two groups is shown on the far-right column


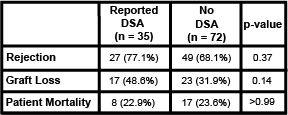


**Supplementary Table 3:** Summary of the median, range, and values for the 20^th^ and 80^th^ percentiles of antibody-verified eplet mismatches within antibody-verified HLA class I, class II (-DR_1/3/4/5_, -DQA1/DQB1), HLA-DR_1/3/4/5_, and HLA–DQA1/DQB1.


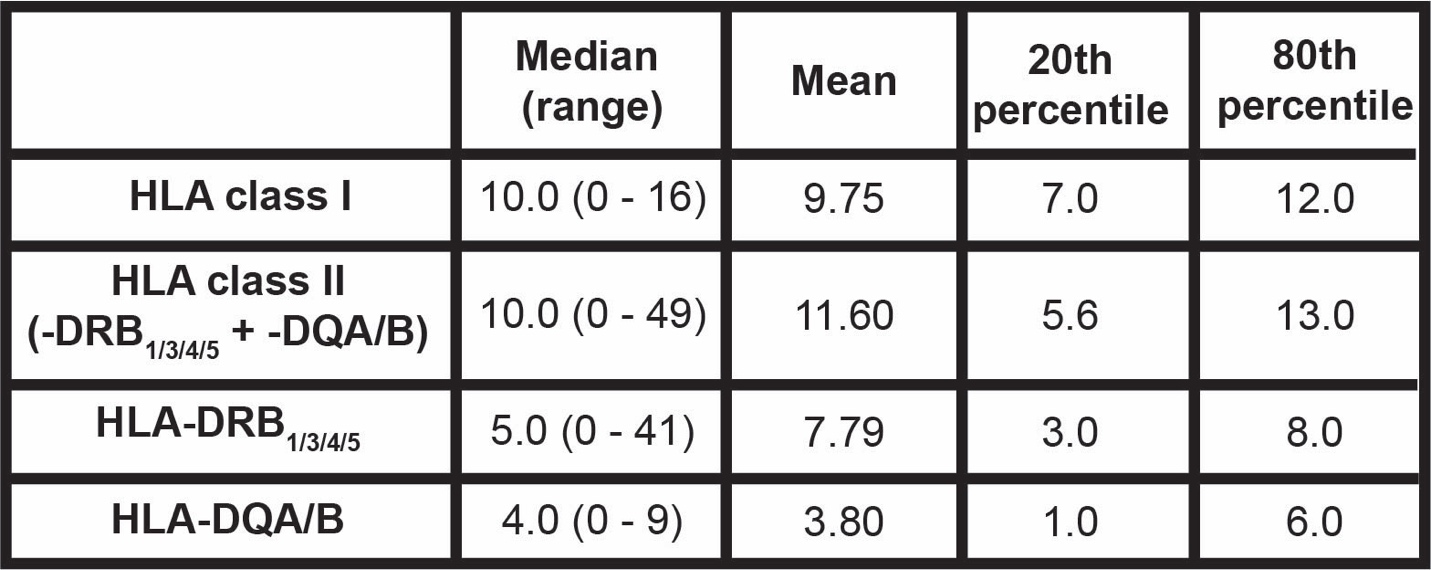


**Supplementary Table 4:** Summary of Mann-Whitney non-parametric t-test p-values when comparing the 20^th^ and 80^th^ percentiles for eplet mismatches within antibody-verified HLA class I, class II (-DR_1/3/4/5_, -DQA1/DQB1), HLA-DR_1/3/4/5_, and HLA–DQA1/DQB1 for DSA development, rejection, graft survival, and patient survival.
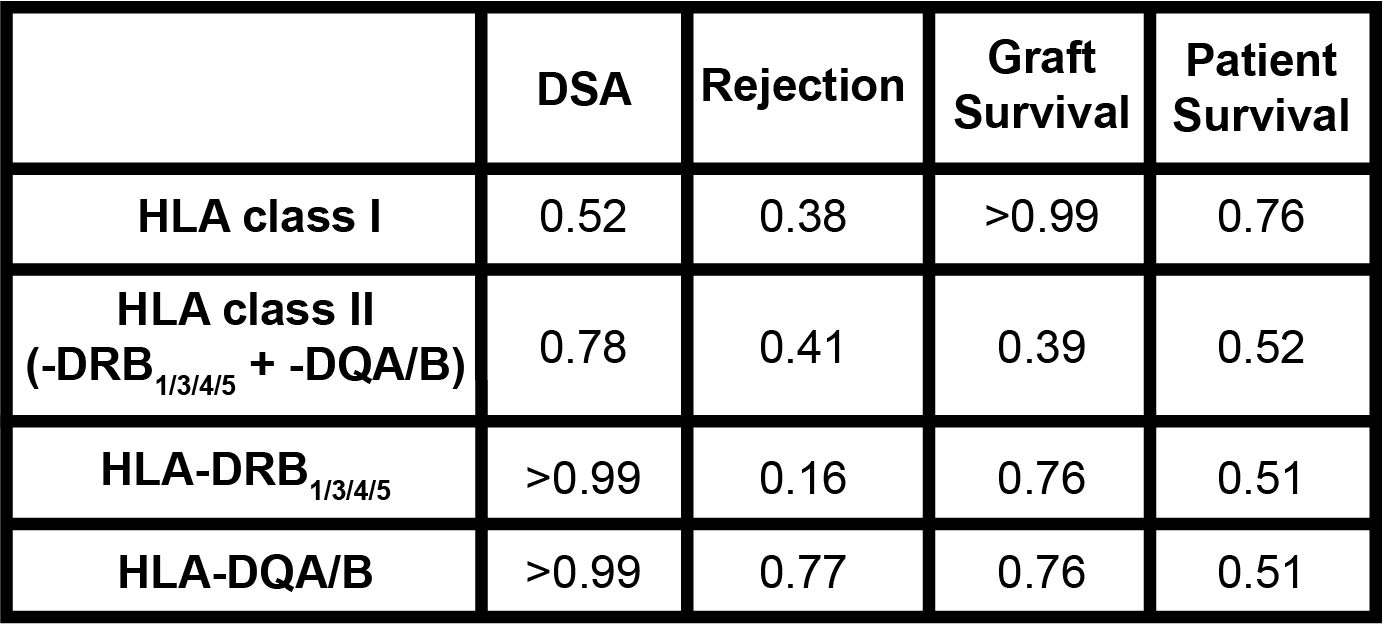

Supplement: Supplementary file 1 — Supplementary material [file mmc1.docx]
